# Optimization of the Production Parameters of Composites from Sugarcane Bagasse and Iron Salts for Use in Dye Adsorption

**DOI:** 10.1155/2019/8173429

**Published:** 2019-07-01

**Authors:** Carine Pereira da Silva, Marluce Oliveira da Guarda Souza, Walter Nei Lopes dos Santos, Laiana Oliveira Bastos Silva

**Affiliations:** ^1^Escola Politécnica, Universidade Federal da Bahia, Salvador, BA 40210-630, Brazil; ^2^Departamento de Ciências Exatas e da Terra, Universidade do Estado da Bahia, Salvador, BA 41195-001, Brazil; ^3^Instituto Federal do Sertão Pernambucano, Petrolina, PE 56316-686, Brazil

## Abstract

In this study, mechanical mixtures of sugarcane bagasse and iron salts (nitrate, acetate, or a mixture of both) were subjected to thermal decomposition for producing iron oxide and carbonaceous composite materials, which were evaluated as adsorbents for removing dyes from water using methylene blue (MB) as a model system. Aiming to optimize the conditions for obtaining composite adsorbents, the Box-Behnken design (BBD) was used to study the effects of mass sugarcane bagasse/mass iron salt, type of mixture of sugarcane bagasse/iron salt, and temperature on the response to be obtained (adsorption capacity, *q*_*e*_) before the execution of the adsorption tests. The synthesized composites were characterized by X-ray diffraction (XRD) analysis, scanning electron microscopy (SEM), and Brunauer-Emmett-Teller (BET) surface area analysis. The relationship between the characteristics of different materials, based on the processing of statistical data and the results of the adsorption tests, helped determine the routes that led to formation of composites with the most suitable properties for the removal of MB dye. Different phases, such as magnetite (Fe_3_O_4_) and/or maghemite (*γ*-Fe_2_O_3_) and iron carbide (Fe_3_C), were formed. The composites that presented the highest *q*_*e*_ values were SB/IN 1:1 (600°C) and SB/IN-IA 1:2 (400°C). The first, which contained iron carbide (Fe_3_C), according to the XRD results, also showed larger BET surface area than the other composites. These properties may have contributed to the higher MB adsorption efficiency of this material in aqueous medium. The sample SB/IN-IA 1:2 (400°C) had lower specific area and was composed of magnetite and/or maghemite phases. In this case, the high *q*_*e*_ was probably associated with the surface properties promoted by combination with the carbonaceous material, favoring interactions with MB.

## 1. Introduction

The textile industry is one of the main industrial sectors responsible for the contamination of natural waters [1]. In this context, the environmental regulations dealing with the effluents generated by this sector, and the scarcity of water in different parts of the world, forced the industry to review, restructure, and reduce its water consumption and the generation of toxic effluents. The textile industry is one of the main contributors to water pollution due to excessive water consumption and enormous discharge of effluents with high polluting potential. These effluents mainly contain highly organic matter such as dyes [2, 3].

Dyes not only affect the aesthetic merit of water bodies but also can reduce the penetration of light (and consequently photosynthesis) and the concentration of oxygen in the water. A wide variety of dyes—azo, anthraquinone, thiazine, etc.—and their degradation products (e.g., aromatic amines) are highly toxic to animals and humans, and many of these products are considered mutagenic and carcinogenic [4–6].

In addition, methylene blue (MB) is one of the most widely used dyes as a biological dye, cyanide antidote, and redox indicator. It is also used in other areas such as the printing and dyeing of fabrics [7, 8]. Although this dye is not strongly hazardous, its harmful effects on aquatic organisms have been well documented [7]. It is a cationic, aromatic, and heterocyclic thiazine dye that shows an intense blue color in the oxidized state and is colorless in its reduced form [9].

Residual waters containing dyes are usually the most difficult to treat by conventional methods owing to their complex aromatic structures that make them thermally and physicochemically stable [10, 11]. Thus, the treatment of effluents before their disposal in water resources is of extreme importance, because if the textile effluent is completely treated, there is a possibility of reuse for other purposes of up to 90% [3].

Among the treatment processes employed, adsorption has gained considerable research attention; it is a promising method owing to its effectiveness and complete removal of organic dyes, as well as easy separation of the adsorbent from the aqueous phase after the final treatment [12]. According to Nascimento et al. [13], adsorption is a mass transfer process that allows the separation of existing components in liquid or gaseous phases based on their concentration on the surface of a solid with suitable properties for adsorption. The adsorbate is deposited on the solid surface, the latter being called the adsorbent.

The search for suitable adsorbent solids has motivated research for the development of new materials with high adsorptive capacity [14]. In this context, several sources of biomass have been found that can be used as bioadsorbents or in the production of materials with adsorptive properties, such as activated carbon and composites. Examples include sugarcane bagasse and other agroindustrial residues, which are mainly composed of cellulose, hemicellulose, and lignin [15].

Pure sugarcane bagasse and that modified with sulfuric acid, as well as activated carbon obtained from sugarcane bagasse, were used. These are good adsorbents owing to their high maximum adsorption capacity [16, 17]. MB was also studied as a contaminant model to evaluate the capacity of pure bagasse modified with CaCl_2_ and with NaOH. A greater adsorptive capacity was found for bagasse treated with NaOH, mainly owing to the complexity of its surface after chemical treatment [18].

Iron oxides are materials with excellent magnetic, electrical, physicochemical, and morphological properties, which make them suitable adsorbents for the removal of pollutants in aqueous media [19]. There are several studies available in the literature on the use of iron oxides as adsorbents owing to not only their low cost but also their high stability, nontoxicity to the environment, high adsorption capacity, and different forms of use, especially when combined with other materials with different properties [20].

Among the several proposed applications of iron oxides in the adsorption of MB dye in aqueous solution, the use of goethite nanoparticles [21], as well as nanospheres formed by a combination of different iron oxides (hematite and magnetite) [22], achieved significant results in the adsorption of MB according to the conditions applied in experiments. Nanoparticles of magnetite (*γ*-Fe_2_O_3_) and magnetite (Fe_3_O_4_), in addition to nanoparticles of magnetite modified with tannic acid, were also used as adsorbents to remove MB in an aqueous solution [23–25].

Other materials with the potential for use in adsorption can be obtained from the combination of iron oxides and carbonaceous material. This forms composites that may present important scientific and technological progress owing to the combination of the adsorption properties of the activated carbon with the properties of iron oxides dispersed on its surface [26].

The production of these composites can be optimized using experimental designs such as the Box-Behnken design (BBD), which is considered a class of second-order rotary (or rotational) drawings based on an incomplete three-level factorial design [27, 28]. In addition to the application of chemometric tools in the optimization of analytical methods, experimental designs have also been applied in the development of new products. This has allowed for the evaluation of alternative materials, the selection of factors so that products can be applied in a wide variety of experimental conditions, and the creation of a product of great performance in a determined process. Therefore, the use of experimental design in this area can result in products that are easier to obtain and exhibit higher performance and reliability, lower cost, and shorter production time [29].

In the meantime, this work had the objective of producing composites with properties suitable for use as adsorbents via the thermal decomposition of sugarcane bagasse and iron salts (nitrate and/or iron acetate). The Box-Behnken design (BBD) was used to optimize the adsorbent-obtaining conditions by studying the effects of the following variables: sugarcane mass/iron salt mass, type of mixture sugarcane bagasse/iron salt and temperature on the response to be obtained (adsorption capacity, *q*_*e*_), and using the adsorption of MB dye as a model system.

## 2. Materials and Methods

### 2.1. Reagents and Materials

The sugarcane bagasse used was obtained from a commercial producer of sugarcane juice, located in the city of Salvador, BA, and the sugarcane was cultivated in the municipality of São Felipe, BA. Iron(II) acetate (Sigma-Aldrich) and commercial iron(III) nitrate nonahydrate (Vetec) were used as precursor salts to obtain iron oxides and were used directly without treatment. The methylene blue (MB) (Synth) solutions in the desired concentration range were made using ultrapure water (electric conductivity 0.05 *μ*S/cm).

### 2.2. Pretreatment of Sugarcane Bagasse

Initially, the sugarcane bagasse (SB) underwent a pretreatment. To this end, this biomass was washed with running water several times to remove the coarser and superficial impurities. Then, it was treated according to the adapted procedure described by Brandão et al. [30], in which the bagasse fibers were immersed in a distilled water bath for 72 hours, with the water exchange done every 12 hours. After this time, the fibers were immersed in an ultrapure water bath for approximately 12 hours. Afterwards, the bagasse was dried in an oven at 60°C until reaching a constant mass, which lasted for approximately 72 hours, and was later crushed and sieved at 30 mesh in a knife mill.

### 2.3. Optimization: Box-Behnken Design (BBD)

In the optimization stage of the composite production, the response surface methodology (RSM) was used, using the BBD. The independent variables studied (factors) were (1) the ratio of the masses of sugarcane bagasse and iron salts used in the production of the composites; (2) the type of mixture used in the production of composites, using sugarcane bagasse and different iron salts; and (3) the temperature. The investigated response (dependent variable) was the adsorption capacity at equilibrium (q_e_, mg g^−1^) of the methylene blue dye (MB) from the adsorption process. STATISTICA® software version 7.0 was used to obtain the planning matrix, the analysis of variance (ANOVA) table, and the response surfaces.  Table 1 shows the levels studied for each of the factors chosen in the optimization stage.

### 2.4. Preparation of Composites

The composites were prepared from the physical mixture between the sugarcane bagasse (SB) and the iron salts: iron nitrate (IN) and/or iron acetate (IA), in proportions (mass/mass) 1:2, 1:1, and 2:1, following a procedure adapted from Gonçalves et al. [31]. After preparation of the precursor mixtures as previously described, they were transferred to quartz cells for further heat treatment in a muffle furnace under the following conditions: rate of 10°C min^−1^; nitrogen flow of 80 mL min^−1^; at 400, 500, or 600°C for 2 h. The obtained materials were sieved through an 80-mesh sieve to guarantee granulometric homogeneity. The composites were named according to the ratio of SB to iron salts (IN and IA) in the mixture and the calcination temperature: SB/IA 1:2 (500°C), SB/IA 1:1 (400°C), SB/IA 1:1 (600°C), SB/IA 2:1 (500°C), SB/IN 1:2 (500°C), SB/IN 1:1 (400°C), SB/IN 1:1 (600°C), SB/IN 2:1 (500°C), SB/IN-IA 2:1 (400°C), SB/IN-IA 2:1 (600°C), SB/IN-IA 1:2 (400°C), SB/IN-IA 1:2 (600°C), SB/IN-IA 1:1 (500°C) 13, SB/IN-IA 1:1 (500°C) 14, and e SB/IN-IA 1:1 (500°C) 15, with the last three being the central points (CP) of the BBD matrix.

### 2.5. Characterization

The most efficient composites for adsorption of MB in aqueous media were characterized by X-ray diffraction (XRD, Shimadzu Model XRD-6000), with an angular variation of 10-80° (2*θ*), copper radiation (Cu K*α*, *λ* = 1.5406 Å), an applied current of 30 mA, and an acceleration voltage of 40 kV. The scanning speed employed was 2° min^−1^. The scanning electron microscopy (SEM) analysis was performed using Tescan brand equipment, model VEGA 3 LMU. To do so, the samples were placed in a carbon fiber covered stub and then metallized in Quorum Technologies equipment, model Q150 RES, where the coatings were made by deposition of metallic ions of gold. The micrographs were obtained using an acceleration voltage of 10 kV and a secondary electron detector. The nitrogen adsorption/desorption isotherms at −196°C were obtained using a Quantachrome equipment, version 11.03. The Brunauer-Emmett-Teller (BET) method was used to determine the specific surface areas of the synthesized composites.

### 2.6. Adsorption Tests

Experiments to evaluate the adsorption capacities of the synthesized composites were done in batches. For this, 100 mg of each adsorbent was added to 100 mL of the dye solution in the following concentrations: 1.0, 2.0, 3.0, 4.0, 5.0, and 6.0 mg L^−1^. The resulting systems were kept under agitation (120 rpm) for 60 min. Then, they were then centrifuged for 15 min. All adsorption tests were performed at a temperature of 23±2°C. Finally, the supernatant was analyzed by Ultraviolet-Visible Spectrometry (Biospectro, SP-22) to determine the remaining concentration of MB dye (C_e_) at a wavelength of 664 nm. The amount of adsorbed material (*q*_*e*_ in mg g^−1^) is determined by

(1) $$\begin{array}{c} q_{e}=\frac{\left(C_{0}-C_{e}\right)V}{m}, \end{array}$$

where *C*_0_ and *C*_*e*_ are the initial and remaining concentrations of MB, respectively;* V* is the volume of solution; and *m* is the mass of the adsorbents.

## 3. Results and Discussion

### 3.1. Statistical Analysis and Response Surface Methodology

Response surface methodology (RSM) was the optimization technique employed. The Box-Behnken design (BBD) was used to obtain a polynomial regression equation to determine the interactions of the selected variables by identifying the significant effects [32, 33]. The optimum region of the surface investigated in the adsorption of MB at an initial concentration of 6.0 mg L^−1^ was obtained.

Central point (CP) triplicates were made to estimate the pure error associated with the repetitions [33, 34]. A total of 15 tests were performed in the optimization stage of the variables to produce efficient composites in the adsorption of MB dye. The BBD matrix of the independent variables (at their real and coded levels) and the observed response values can be seen in Table 2.

It is possible to infer from the values of the responses that, for each of the materials prepared from the combinations of the factors evaluated, the adsorption capacity (*q*_*e*_) ranged from 0.0 to 5.84 mg g^−1^ considering the characteristics of each sample/dye system. However, for tests 7, 8, and 12 (Table 2), the values of *q*_*e*_ were assigned as zero owing to changes in the characteristics of the final solutions after the adsorption process of the MB. These changes prevented readings from being carried out with clarity in the UV-Vis spectrophotometer.

From the results shown in Tables 3 and 4, it can be observed that the model employed did not show a lack of fit (i.e.,* p* > 0.05). In addition, the linear term of the factor type of mixture sugarcane bagasse/iron salt (*x*_2_), as well as the interaction term between this factor and the temperature (*x*_3_), is significant. In addition, the pure error is relatively small.

The analysis of variance can be determined from the equation* SS*_*T*_* = SS*_*R*_* + SS*_*r*_, which represents the quadratic sum around the mean (*SS*_*T*_) and the quadratic sum owing to the regression (*SS*_*R*_) and residual quadratic sum (*SSr*), respectively. Obtaining the value of the ratio between* SS*_*R*_ and* SS*_*T*_, we have the coefficient of determination of the model (*R*^2^) [32]. The closer the value of *R*^2^ is to 1, the better the model fits to the observed responses (2). Thus, by calculating the quadratic sum values (total and owing to regression) from the observed responses and those predicted by the model, we have the following value:

(2) $$\begin{array}{c} R^{2}=\frac{{SS}_{R}}{{SS}_{T}}=\frac{\sum {\left({\hat{y}}_{i}-\overset{-}{y}\right)}^{2}}{\sum {\left(y_{i}-\overset{-}{y}\right)}^{2}}=\frac{37.693}{59.440}=0.6341, \end{array}$$

where

*SS*_*R*_ is the deviation of the prediction made by the model to point ${\hat{y}}_{i}$ in relation to the mean $\overset{-}{y}$.

*SS*_*T*_ is the deviation of an individual response *y*_*i*_ in relation to the mean of the responses.

*SS*_*r*_ is the deviation of the response observed in relation to the response predicted by the model.

According to the *R*^2^ value obtained, only 63.41% of the total variation around the mean can be explained by the regression, and 36.59% of this is owing to the residuals left by the model. That is, there are differences between the responses observed and those predicted by the model. On the other hand, this value should not be compared to 100% owing to the contribution of the pure error [32]. Therefore, the maximum percentage of the explainable variation can be obtained as follows:

(3) %  máxima  de  variação  explicável=SST−SSepSST

(4) %  máxima  de  variação  explicável=59.440−0.73959.440=0.9875×100=98.75%.

The residual quadratic sum (*SS*_*r*_) can be calculated by the sum of the squares of the differences between the observed value (*y*_*i*_) and the predicted value (${\hat{y}}_{i}$), that is,* SS*_*r*_ = $\sum {\left(y_{i}-{\hat{y}}_{i}\right)}^{2}$, and can be represented by* SS*_*T*_* = SS*_*R*_* + SS*_*r*_. However, the residual quadratic sum left by the model can be decomposed into two parts: (1) caused by random errors and (2) in relation to the model's lack of fit [32, 34]. That is,

(5) Residual  SS=SS  owing  to  pure  error+SS  owing  to  lack  of  adjustmentor  SSr=SSep+SSfaj.

Another parameter can also be used to evaluate the lack of fit of a model in relation to the observed responses based on the *F* test (Fisher distribution) of the ratio between the media of the square owing to the lack of fit (*MS*_*lof*_) and the media of the square owing to the pure error (*MS*_*pe*_) [32]. The values of each of these media of the squares are listed in Tables 3 and 4, and the value of the ratio *MS*_*lof*_/*MS*_*pe*_ is 18.93 (as in (6)). Compared to the value of *F*_3,2_ = 19.16 (95% confidence level), there is no evidence of a lack of fit of the model since *MS*_*lof*_/*MS*_*pe*_ < *F*_3,2_.

(6) $$\begin{array}{c} \frac{{MS}_{faj}}{{MS}_{ep}}=\frac{7.003}{0.370}=18.93, \end{array}$$

where

*MS*_*faj*_ is the quadratic mean owing to lack of fit.

*MS*_*ep*_ is the quadratic mean owing to pure error.

Considering the interpretation of the adjustment of the model to the experimental results of the adsorption capacity (*q*_*e*_) obtained, the empirical model for the adsorption capacity of the MB by the adsorbents prepared from the BBD can be described by the following second-order polynomial equation:

(7) qe=3.756−0.756x1−1.081x2−0.443x3−1.140x12−1.325x22−0.619x32+0.589x1x2+0.699x1x3−1.383x2x3.

This mathematical equation describes the behavior of the response (*q*_*e*_) according to the levels (or classes) of the studied factors [34], which are as follows: mass of sugarcane bagasse/mass of iron salt (*x*_1_), type of mixture of sugarcane bagasse/iron salt (*x*_2_), and temperature (*x*_3_). In practice, (2) can be used to predict the experimental conditions that will result in a higher *q*_*e*_. Then, the region contains a maximum point located at approximately *x*_1_ = − 0.49, *x*_2_ = −0.45, and *x*_3_ = − 0.13. For the factors *x*_1_ and *x*_2_, the values at the maximum are close to levels -1 (1:2 and SB/IN, resp.) and 0 (1:1 and SB/IN-IA, resp.), while for x_3_, the real value corresponds to a temperature of 487°C.

**Figure 1:**
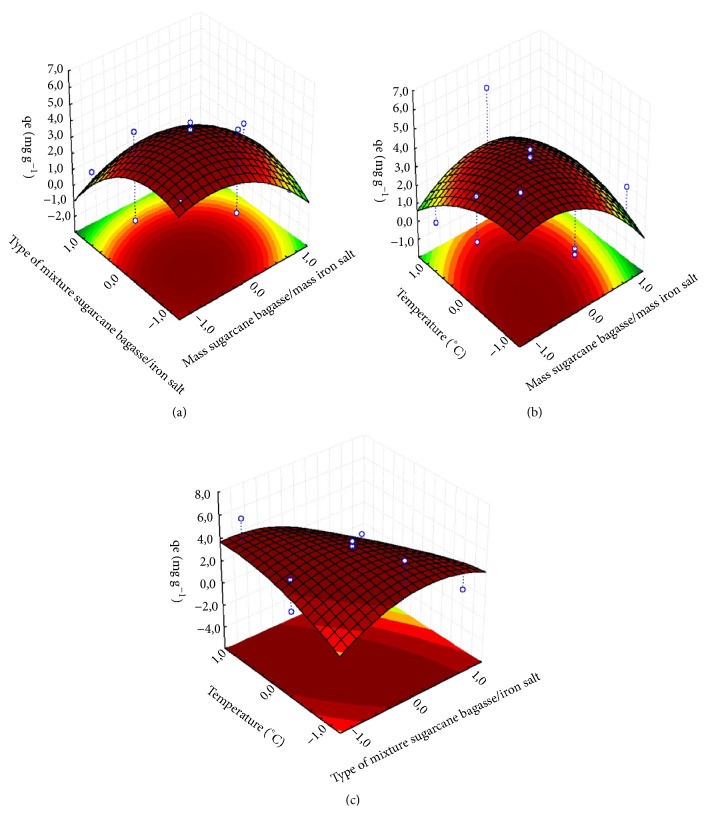
Response surfaces for *q*_*e*_ as a function of (a) mass SB/mass IS and type of mixture SB/IS, (b) mass SB/mass IS and temperature, and (c) type of mixture of SB/IS and temperature. SB: sugarcane bagasse; IS: iron salt.

The response surfaces generated by (2) are shown in Figure 1. The equation of the fitted model can provide initial information about the geometric nature of the response surfaces based on the signals and magnitudes of the quadratic coefficients of the second-order polynomial function obtained. In this context, since all quadratic coefficients are negative (*x*_*1*_^2^,* x*_*2*_^2^, and* x*_*3*_^2^ in (2)), the function may have a maximum, and the obtained response surfaces should exhibit maximum regions within the investigated experimental domain [28, 32, 34].

### 3.2. Effects of Factors Studied

The three-dimensional response surfaces are studied as a function of two factors, while the other is maintained at a fixed level. This is done in order to identify and interpret the relationship between the main and interaction effects of these two factors [34]. The effects of the independent variables analyzed at their respective coded levels (-1, 0, 1) on the *q*_*e*_ of MB will be discussed in the following sections based on the response surfaces (Figure 1).

#### 3.2.1. Effect of Ratio Mass Sugarcane Bagasse/Mas Iron Salt (*x*_1_)

In analyzing Figures 1(a) and 1(b), it is observed that higher adsorption capacities (*q*_*e*_) were obtained at proportions (mass/mass) near the lower (1:2) and central (1:1) levels for all factors, i.e., the best results for the removal of MB in an aqueous medium were obtained when the mass of sugarcane bagasse (SB) was equal to or less than that of the iron salts (iron nitrate and/or iron acetate) in the lower and central levels of the factor types of mixture sugarcane bagasse/iron salt (SB/IN and SB/IN-IA, resp.) and temperature (400°C and 500°C, resp.).

There is a direct relationship between the response surfaces with the Box-Behnken design (BBD) (Table 2); in relation to the mass factor of the sugarcane bagasse/iron salt mass (x_1_), in the same type of bagasse/iron salt mixture (SB/IN) and at the same temperature (500°C), the change in the bagasse ratio (SB) and iron nitrate of 1:2 (run 1) to 2:1 (run 2) was sufficient to promote a considerable decrease in MB adsorption capacity of 3.32 mg g^−1^ for 0.51 mg g^−1^. This can be explained by considering the properties of the obtained materials and will be discussed by reviewing the characterization techniques used.

In comparing runs 3 and 4, a change in the ratio between sugarcane bagasse (SB) and iron acetate (IA) from 1:2 to 2:1 in the same temperature range (500°C) did not promote significant changes in the adsorption capacity of the MB. This is because the values of *q*_*e*_ were very small when compared to the values obtained in the other tests performed. Thus, very similar characteristics among the samples, despite the difference in the proportion of the precursor mixtures before heat treatment in the temperature range of 500°C, may be the main explanation for the lower adsorption capacity of the methylene blue.

In relation to tests 5 and 6, for the thermally treated SB/IN-IA mixture at 400°C, there was a significant decrease in the adsorption capacity of the dye under study when the ratio of cane bagasse (SB) to iron salts (IN-IA) ranged from 1:2 to 2:1. In this context, a higher value of *q*_*e*_ was obtained for test 5 (5.39 mg g^−1^) compared to experiment 6 (2.60 mg g^−1^), suggesting changes in the properties of the prepared composites when the ratio of cane bagasse (BC) to the nitrate and iron acetate salts was modified from 1:2 to 2:1.

#### 3.2.2. Effect of Type of Mixture of Sugarcane Bagasse/Iron Salt (*x*_2_)

According to the ANOVA Table (Table 4), the factor type of the sugarcane bagasse/iron salt mixture was significant (*p *< 0.05 and a high value of* F*), suggesting that this is the parameter that most influences the adsorption capacity of the methylene blue dye. This was studied in three categories: SB/IN (-1), SB/IN-IA (0), and SB/IA (1).

From the response surface of Figure 1(a), it can be concluded that, in relation to the factor of mass sugarcane bagasse/mass iron salt (*x*_1_), higher *q*_*e*_ values were obtained in the categories near the central (SB/IN-IA) and lower (SB/IN) points. Analyzing Figure 1(c) (type of mixture sugarcane bagasse/iron salt plotted against temperature), it is observed that a greater adsorption of the MB was obtained in categories close to the central point (SB/IN-IA) for the three temperature levels investigated (400, 500, and 600°C).

By making a relation between the response surfaces represented by Figures 1(a) and 1(c) and the BBD matrix (Table 2), it can be observed that, for the same proportion (mass/mass) of sugarcane bagasse and iron salt (1:2) and the same temperature range (500°C), a change in the type of mixture [SB/IN (test 1) and SB/IA (test 3)] is sufficient to promote a decrease in the adsorption capacity of MB from 3.32 mg g^−1^ to 0.89 mg g^−1^. There were differences in the adsorption mechanism of these materials during the removal of MB in aqueous solution. These differences resulted from the properties and characteristics of the adsorbent composites as a consequence of the use of different precursors of iron oxides (nitrate and iron acetate).

Comparing tests 2 and 4, it was found that, for a ratio of 2:1 of the precursor mixture and a temperature of 500°C, the change in the type of mixture did not significantly interfere with the values of *q*_*e*_. This occurred because the results were significantly lower for both assays, and the samples were then considered as inefficient for MB adsorption. Even with different iron salts, a greater amount of sugarcane bagasse (SB) present in the precursor mixture was decisive in promoting modifications in the properties of the prepared samples and consequently their efficiency in the adsorption process.

However, for experiments 9 and 10, while maintaining the proportion between the components of the mixture at 1:1 and a temperature at which the samples were thermally treated at 400°C, when changing the type of SB/IN blend to SB/IA, no significant changes in the adsorption capacity of the MB were observed. This was different from the results obtained when comparing runs 11 and 12, in which composites formed by mixtures in proportions of 1:1 at 600°C presented a high adsorption capacity of MB when the mixture was between SB and IN (5.84 mg g^−1^) and a supposedly nonexistent adsorption capacity when the mixture was SB/IA (0.0 mg g^−1^).

On the other hand, comparing tests 9 and 11 with experiments 10 and 12, it can be observed that the increase in temperature (from 400 to 600°C) only favored the adsorption of MB when working with SB/IN mixtures at 1:1, as the adsorption capacity increased at equilibrium from 0.86 to 5.84 mg g^−1^. By contrast, when working with SB/IA mixtures also at a ratio of 1:1, a temperature increase slightly affected the adsorption capacity. This obtained lower values for the experimental conditions, evidencing the interaction between the factors.

#### 3.2.3. Effect of Temperature (*x*_3_)

According to the ANOVA Table (Table 4), the interaction between the temperature (*x*_3_) and the type of mixture of sugarcane bagasse/iron salt (*x*_2_) was significant. Thus, changes in these two factors together promoted significant changes in the adsorption capacity of MB in aqueous solution.

By analyzing the response surface of Figure 1(b), it can be inferred that higher adsorption capacities in the equilibrium (*q*_*e*_) were obtained for values between the intermediate (500°C) and lower (400°C) levels when the levels of the factor of mass sugarcane bagasse/mass iron salt were situated between -1 (1:2) and 0 (1:1). However, Figure 1(c) shows that a higher *q*_*e*_ was obtained at higher temperatures [central (500°C) and higher (600°C)] when the factor type of the mixture of sugarcane bagasse/iron salt was located between the lower (SB/IN) and central (SB/IN-IA) levels.

By making a connection between the response surfaces obtained as a function of temperature and the results of the BBD matrix (Table 2), it is possible to make predictions about the effects of temperature changes in relation to the studied response (adsorption capacity *q*_*e*_). Therefore, when comparing tests 5 and 7, it can be seen that, for composites formed from SB/IN-IA 1:2 mixtures, when they were thermally treated at 400°C, the adsorption capacity was much higher (5.39 mg g^−1^) compared with the response obtained when the treatment temperature was 600°C (0.00 mg g^−1^).

In experiments 6 and 8, from the SB/IN-IA 2:1 mixtures, raising the temperature from 400 to 600°C promoted a significant reduction in the dye adsorption capacity of 2.60 mg g^−1^ (test 6) to 0.00 mg g^−1^ (test 8) owing to the same behavior obtained in the comparison of tests 5 and 7. On the other hand, comparing experiments 9 and 11, it is observed that, for SB/IN 1:1 mixtures, the treatment temperature increased from 400 to 600°C, promoting an increasing adsorption capacity of MB from 0.86 mg g^−1^ (test 9) to 5.84 mg g^−1^ (test 11). As for runs 10 and 12, unlike previous discussions of the results for SB/IA 1:1 mixtures, the increase in temperature did not promote significant changes in the adsorption capacity of the synthesized composites since both samples presented low values of adsorption capacity (*q*_*e*_).

### 3.3. Characterization of Adsorbent Composites

BBD was used to obtain the best conditions to produce composites (formed from sugarcane bagasse and iron salts) that may be efficient adsorbents when using MB as the model system. However, to complement the BBD study, the properties and characteristics of the most promising systems were determined in order to contribute to the discussion of the results obtained in the statistical analysis. For this, characterization of the samples was done using the following techniques on the surface area: XRD, SEM, and BET surface area.

**Figure 2:**
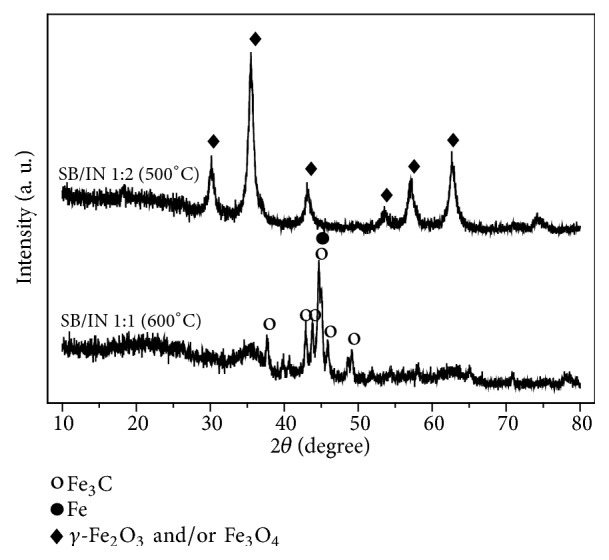
X-ray diffractograms of composites SB/IN 1:2 (500°C) and SB/IN 1:1 (600°C).

**Figure 3:**
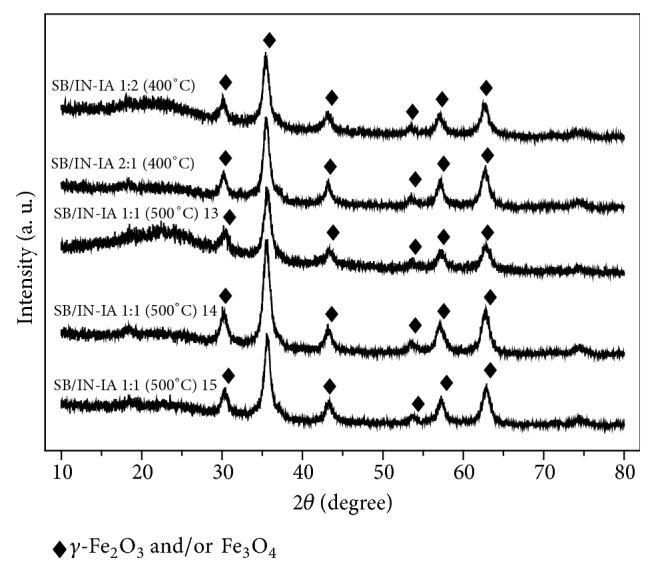
X-ray diffraction patterns of composites SB/IN-IA 1:2 (400°C), SB/IN-IA (400°C), SB/IN-IA 1:1 (500°C) 13, SB/IN-IA 1:1 (500°C) 14, and SB/IN-IA 1:1 (500°C) 15.

Diffractograms of the most promising adsorbent composites for the adsorption of MB in an aqueous medium are shown in Figures 2 and 3. The *q*_*e*_ values for all of these materials are listed in Table 2.

The diffractogram of the composite SB/IN 1:2 (500°C) is characteristic of the magnetite phase (Fe_3_O_4_) (JCPDS file no. 01-088-0866) and/or maghemite phase (*γ*-Fe_2_O_3_) (JCPDS file no. 00-039- 1346). The XRD technique was limited to differentiating the two phases of iron oxides [35].

The diffractogram of the composite SB/IN 1:1 (600°C) (Figure 2) is characteristic of iron carbide (Fe_3_C) (JCPDS file no. 00-035-0772) according to the peaks observed at 2*θ*: 37.70°, 42.86°, 43.76°, 45.92°, and 49.14°. A peak is also seen at 2*θ*: 44.68°, assigned to Fe^0^ (JCPDS file no. 01-071-4648) [36–38]. Iron carbide is often obtained with unconverted Fe^0^ or a carbonaceous deposit [39]. It is also observed that iron oxide (mainly magnetite) was converted into iron carbide (Fe_3_C), evidenced by the complete disappearance of crystalline oxide peaks in the diffractogram of the sample obtained at 600°C [40].

The diffractograms of composites SB/IN-IA 1:2 (400°C) and SB/IN-IA 2:1 (400°C) (Figure 3) show that the iron oxide phase present in these materials can be maghemite (*γ*-Fe_2_O_3_) (JCPDS file no. 00-039-1346), magnetite (Fe_3_O_4_) (JCPDS file no. 01-088-0866), or a mixture of the two phases. These are formed by the thermal decomposition of iron (III) nitrate and iron (II) acetate together with the sugarcane bagasse (SB).

The diffractograms of the samples comprising the central points (CP) [SB/IN-IA 1:1 (500°C) 13, SB/IN-IA (500°C) 14, and SB/IN-IA 1:1 (500°C) 15] are very similar (Figure 3), which was expected since these composites were produced under the same experimental conditions. In these materials, the iron oxide phase present may be maghemite (*γ*-Fe_2_O_3_) (JCPDS file no. 00-039-1346) and/or magnetite (Fe_3_O_4_) (JCPDS file no. 01-088-0866), showing peaks at 2*θ* = 30.23°, 35.45°, 43.17°, 53.61°, 57.17°, 62.72°, and 74.19°.

**Figure 4:**
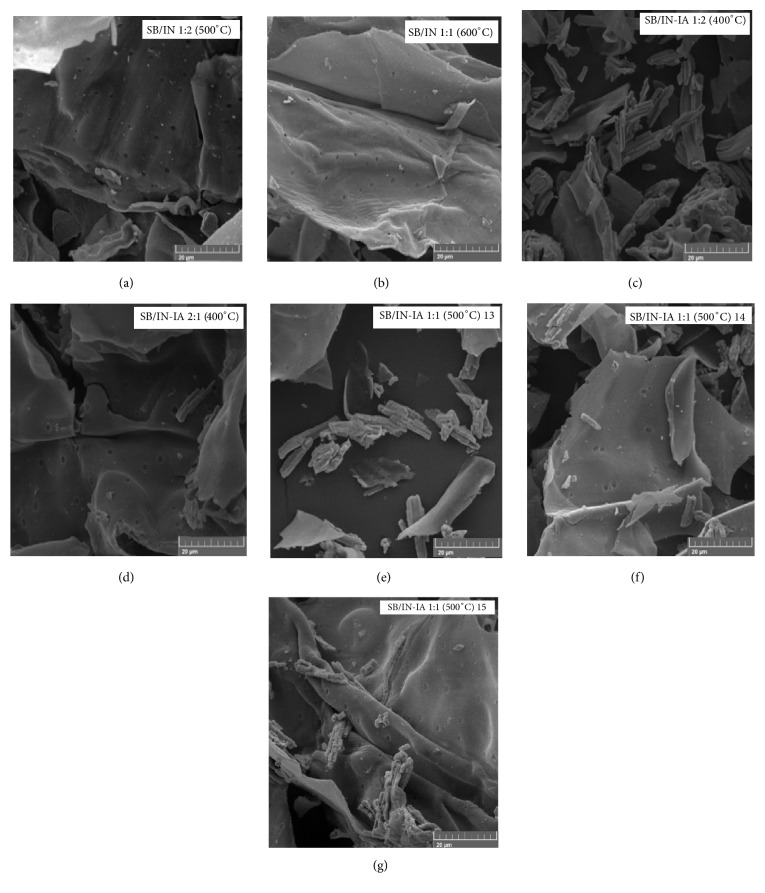
SEM images for composites (a) SB/IN 1:2 (500°C), (b) SB/IN 1:1 (600°C), (c) SB/IN-IA 1:2 (400°C), (d) SB/IN-IA 2:1 (400°C), (e) SB/IN-IA 1:1 (500°C) 13, (f) SB/IN-IA 1:1 (500°C) 14, and (g) SB/IN-IA 1:1 (500°C) 15.

Analyzing the SEM images, the composite SB/IN 1:2 (500°C) [Figure 4(a)] shows leaf-shaped particles. This is similar to the morphology of sugarcane bagasse (SB) calcined at 500°C. Pores on the external surface were also observed, characterizing the carbonaceous material originating from the carbonization of SB. However, it is not possible to clearly distinguish the morphology associated with iron oxides formed from the precursor salt iron (III) nitrate. In addition, the SB/IN 1:1 (600°C) material shows a smooth-looking carbonaceous matrix-like sheet morphology [41, 42], as can be seen in the SEM images of Figure 4(b). In addition, the external surface of the leaves has pores, seen clearly in the image in question.

The micrographs of composites SB/IN-IA 1:2 (400°C) and SB/IN-IA 2:1 (400°C) are shown in Figures 4(c) and 4(d), respectively, and are quite similar. Thus, based on the SEM images, a rather heterogeneous morphology is identified, with the presence of leaves of smooth carbonaceous material and pores, obtained by decomposition of SB. This morphology is more obvious in the second composite [Figure 4(d)]. In addition, the iron oxide particles (which may be from the magnetite) in the form of rods may be related to the thermal decomposition of iron(II) acetate, seen more clearly in the composite SB/IN-IA 1:2 (400°C) [Figure 4(c)].

As for the SEM images of the central points, shown in Figures 4(e)–4(g), it can be seen that porous sheets of carbonaceous material from the heat treatment of the sugarcane bagasse (SB) are present, as well as iron oxide particles in the form of rods.

Table 5 lists the BET surface area values of the most promising composites for use in the study of the adsorption of dyes, identified from the results of the BBD matrix (Table 2).

The composite with the largest surface area of 223 m^2 ^g^−1^ was SB/IN 1:1 (600°C). According to the XRD results, this material is mainly formed by iron carbide (Fe_3_C) and was the sample that presented a higher adsorption capacity (*q*_*e*_) of mg g^−1^.

In the other composites of carbonaceous material/iron oxide whose oxide phases are maghemite and/or magnetite, a direct relation between the area and the adsorption capacity was not observed because the second most promising composite (*q*_*e*_ = 5.39 mg g^−1^) presented the smallest specific area (53 m^2 ^g^−1^). This indicates that the other properties are relevant. In this way, it can be inferred that, among those whose phases are magnetite and/or maghemite, a higher *q*_*e*_ of a sample with a lower specific area can be the result of a more favorable surface for the adsorption process, promoted by the combination with carbonaceous material and interaction with the MB adsorbate. However, all of the solids shown in Table 5 can be efficiently used in adsorption processes owing to their high *q*_*e*_ values.

## 4. Conclusion

The use of the Box-Behnken design (BBD) to optimize the production conditions of adsorbent composites was efficient and generated a model that fit well with the experimental data for the adsorption capacity at equilibrium (*q*_*e*_). The factor type of mixture sugarcane bagasse/iron salt (x_2_), and the interaction term between x_2_ and the temperature (x_3_), were significant for the system in the experimental domain investigated. The composites that presented the highest *q*_*e*_ values were SB/IN 1:1 (600°C) and SB/IN-IA 1:2 (400°C). The first, which contained iron carbide (Fe_3_C), also showed a larger BET surface area compared to those of the other composites. These properties may have contributed to the higher efficiency of this material in the adsorption of MB dye in an aqueous medium. The sample SB/IN-IA 1:2 (400°C) had a lower specific area, and the phases were magnetite and/or maghemite. In this case, the high *q*_*e*_ was probably associated with the surface properties promoted by its combination with carbonaceous material, favoring interactions with MB adsorbate.

## Figures and Tables

**Table 1:** Factors (independent variables) and their respective levels (or classes) chosen in the optimization stage of the composite production.

Independent variables	Levels/Classes
Mass sugarcane bagasse/mass iron salt	1:2; 1:1; 2:1

Type of mixture sugarcane bagasse/iron salt	Sugarcane bagasse/iron nitrate (SB/IN); Sugarcane bagasse/iron acetate (SB/IA); Sugarcane bagasse/ iron nitrate-iron acetate (SB/IN-IA)

Temperature	400°C; 500°C; 600°C

**Table 2:** Box-Behnken design (BBD) matrix with real and coded values of three factors studied, and response (*q*_*e*_) of each test performed.

Run	Independent Variables			Dependent Variable
	**x**_**1**_ Mass sugarcane bagasse/mass iron salt (g)	**x**_**2**_ Type of mixture sugarcane bagasse/iron salt	**x**_**3**_ Temperature (°C)	**Response (y)** q_e_ (mg g^−1^)
1	1:2 (-1)	SB/IN (-1)	500 (0)	3.32

2	2:1 (1)	SB/IN (-1)	500 (0)	0.51

3	1:2 (-1)	SB/IA (1)	500 (0)	0.89

4	2:1 (1)	SB/IA (1)	500 (0)	0.44

5	1:2 (-1)	SB/IN-IA (0)	400 (-1)	5.39

6	2:1 (1)	SB/IN-IA (0)	400 (-1)	2.60

7	1:2 (-1)	SB/IN-IA (0)	600 (1)	0.00

8	2:1 (1)	SB/IN-IA (0)	600 (1)	0.00

9	1:1 (0)	SB/IN (-1)	400 (-1)	0.86

10	1:1 (0)	SB/IA (1)	400 (-1)	0.55

11	1:1 (0)	SB/IN (-1)	600 (1)	5.84

12	1:1 (0)	SB/IA (1)	600 (1)	0.00

13 (CP)	1:1 (0)	SB/IN-IA (0)	500 (0)	3.87

14 (CP)	1:1 (0)	SB/IN-IA (0)	500 (0)	3.10

15 (CP)	1:1 (0)	SB/IN-IA (0)	500 (0)	4.30

**Table 3:** Analysis of variance for quadratic model adjusted to data set of Table 2.

Variation source	Sum of the square (SS)	Degree of freedom	Media of the square (MS)
Regression	37.693	9	4.188

Residuals	21.747	5	4.349

Lack of fit	21.008	3	7.003

Pure error	0.739	2	0.370

*Total*	*59.440*	*14*	

**Table 4:** Analysis of variance (ANOVA) for quadratic polynomial model for MB adsorption.

Factors	Sum of the square (SS)	Degree of freedom	Media of the square (MS)	*F*	*p*
Mass sugarcane bagasse/mass iron salt (x_1_)	4.574	1	4.574	12.378	0.072156

Mass sugarcane bagasse/mass iron salt (x_1_^2^)	4.797	1	4.797	12.981	0.069141

Type of mixture sugarcane bagasse/iron salt (x_2_)	9.348	1	9.348	25.299	0.037328

Type of mixture sugarcane bagasse/iron salt (x_2_^2^)	6.478	1	6.478	17.530	0.052585

Temperature (°C) (x_3_)	1.569	1	1.569	4.246	0.175493

Temperature (°C) (x_3_^2^)	1.416	1	1.416	3.832	0.189397

Interaction (x_1_) by (x_2_)	1.385	1	1.385	3.749	0.192465

Interaction (x_1_) by (x_3_)	1.953	1	1.953	5.285	0.148253

Interaction (x_2_) by (x_3_)	7.651	1	7.651	20.705	0.045059

Lack of fit	21.008	3	7.003	18.951	0.050539

Pure error	0.739	2	0.370		

*Total *	*59.440*	*14*			

**Table 5:** BET surface area values of composites with higher adsorption capacities.

Composites	BET surface area (m^2^ g^−1^)	q_e_ (mg g^−1^)
BC/NF 1:2 (500°C)	104	3.32

BC/NF 1:1 (600°C)	223	5.84

BC/NF-AF 1:2 (400°C)	53	5.39

BC/NF-AF 2:1 (400°C)	54	2.60

BC/NF-AF 1:1 (500°C) 13	164	3.87

BC/NF-AF 1:1 (500°C) 14	182	3.10

BC/NF-AF 1:1 (500°C) 15	178	4.30

## Data Availability

The data used to support the findings of this study are included within the article.
